# Genetic modification of Candida maltosa, a non-pathogenic CTG species, reveals EFG1 function

**DOI:** 10.1099/mic.0.001447

**Published:** 2024-03-08

**Authors:** Marco Chávez-Tinoco, Luis F. García-Ortega, Eugenio Mancera

**Affiliations:** 1 Departamento de Ingeniería Genética, Unidad Irapuato, Centro de Investigación y de Estudios Avanzados del Instituto Politécnico Nacional, Irapuato, Mexico

**Keywords:** CUG-Ser1 yeasts, CTG clade, Candida maltosa, pathogenic yeasts, EFG1, non-pathogenic *Candida*

## Abstract

*Candida maltosa* is closely related to important pathogenic *Candida* species, especially *C. tropicalis* and *C. albicans,* but it has been rarely isolated from humans. For this reason, through comparative studies, it could be a powerful model to understand the genetic underpinnings of the pathogenicity of *Candida* species. Here, we generated a cohesive assembly of the *C. maltosa* genome and developed genetic engineering tools that will facilitate studying this species at a molecular level. We used a combination of short and long-read sequencing to build a polished genomic draft composed of 14 Mbp, 45 contigs and close to 5700 genes. This assembly represents a substantial improvement from the currently available sequences that are composed of thousands of contigs. Genomic comparison with *C. albicans* and *C. tropicalis* revealed a substantial reduction in the total number of genes in *C. maltosa*. However, gene loss seems not to be associated to the avirulence of this species given that most genes that have been previously associated with pathogenicity were also present in *C. maltosa*. To be able to edit the genome of * C. maltosa* we generated a set of triple auxotrophic strains so that gene deletions can be performed similarly to what has been routinely done in pathogenic *Candida* species. As a proof of concept, we generated gene knockouts of *EFG1,* a gene that encodes a transcription factor that is essential for filamentation and biofilm formation in *C. albicans* and *C. tropicalis*. Characterization of these mutants showed that Efg1 also plays a role in biofilm formation and filamentous growth in *C. maltosa*, but it seems to be a repressor of filamentation in this species. The genome assembly and auxotrophic mutants developed here are a key step forward to start using *C. maltosa* for comparative and evolutionary studies at a molecular level.

## Data Summary

The raw genome data, the genome assembly and the annotation have been deposited at the NCBI under BioProject ID PRJNA1036158.

## Introduction

**Fig. 1.**
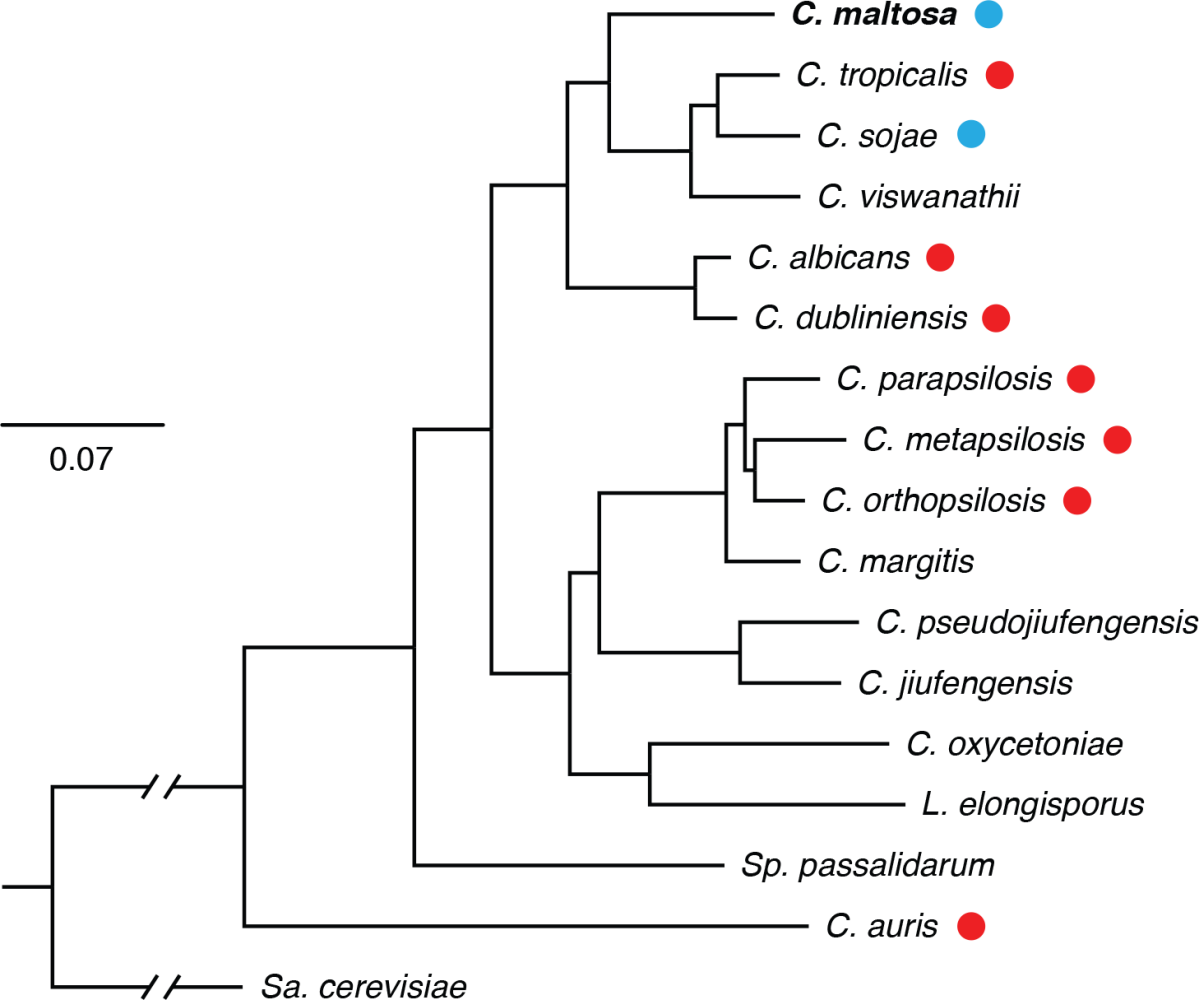
Phylogenetic relationship of *C. maltosa* with its closest CTG species and their association to humans. Maximum-likelihood phylogenetic tree based on the alignment of 569 single copy orthologs that were conserved in all the genomes of the species included (see Methods). Bootstrap support, based on 1000 replicates, was 100 in all cases, but for the node leading to *C. metapsilosis* and *C. orthopsilosis* where it was 97. The genetic-distance scale represents substitutions per nucleotide. Red circles denote pathogenic species while blue circles non-pathogenic ones according to Gabaldon T. *et al.*, (2016). *C. maltosa* was not included by Gabaldon T. *et al.*, (2016) but given previous evidence and the frequency of isolation from humans it can be considered non-pathogenic.

Fungi from the CTG or CUG-Ser1 clade have attracted human attention for centuries given their medical relevance. This group of ascomycetous yeasts received their name because they translate the CUG codon to serine instead of leucine as most other eukaryotes do [1]. Several of the clinically most important species of fungi such as *Candida albicans*, *C. tropicalis*, *C. parapsilosis* and *C. auris* belong to this clade [2,3]. Worldwide, these microorganisms are responsible for ~700 000 candidiasis cases every year, being an important part of the total number of infections caused by fungi and among the top health threats according to the World Health Organization [4,5]. Interestingly, among the members of the CTG clade there are also species that have been very rarely isolated from humans. These species come from a variety of environments, including soil, air, water and other animals such as insects [6,9]. Several of the non-virulent species are closely related to pathogenic species (Fig. 1) and given their phylogenetic position, the ability to colonize humans is thought to have originated repeated times within the clade [10,11]. Due to their phylogenetic proximity, non-virulent CTG species offer a valuable point of comparison to better understand the pathogenicity of these microorganisms.

Not surprisingly, most of the work to understand the molecular mechanisms that are responsible for pathogenicity has been targeted to the virulent species, mostly to *C. albicans.* Research in other virulent species has accelerated in recent years as some have become more common etiological agents. This is especially the case for *C. auris* which is an emerging pathogen of growing concern given its resistance to multiple typical antifungal drugs [12]. On the other hand, considerably less is known about the biology of non-virulent or less-virulent CTG species. Several studies have compared *C. dubliniensis* at a genomic and molecular level to *C. albicans,* since it is phylogenetically its closest relative and considerably less virulent [3,13, 14]. However, *C. dubliniensis* is still considered an inhabitant of the human body. The genome sequences of several non-pathogenic CTG species are available (e.g. *Candida maltosa, C. sojae, C. tenuis, Debaryomyces hansenii* and *Meyerozyma caribbica*) and broad genomic comparisons have helped to generate hypotheses about the pathways involved in virulence [2]. Research in non-pathogenic CTG species has also focused on biotechnological applications, but, overall, it is considerably little what we know about the way these species have adapted to non-human environments.

*C. maltosa* is a CTG species closely related to *C. tropicalis,* one of the most important human pathogens in the clade. It is also phylogenetically close to *C. albicans* and *C. parapsilosis*, which together with *C. tropicalis* account for most human infections caused by CTG species [15]. The first strain of *C. maltosa* was described in 1964 coming from the adhesives of neutralizing tanks used to produce monosodium glutamate in Japan [16]. As far as we know, there are two reports of its isolation from immunocompromised patients and recently it was detected in human intrauterine samples through ITS amplicon sequencing [7,17]. However, the low frequency of isolation from humans suggests that the human body is not part of its core ecological niche, at least not more than it is for other non-pathogenic species such as the baker’s yeast. Furthermore, given the interest in employing *C. maltosa* for industrial processes, extensive testing in human cell cultures and laboratory animal models strongly indicated that it is a non-pathogenic species [7]. Strains of this species often come from industrial settings or from a variety of natural environments associated to anthropogenic activities, especially if enriched with hydrocarbons [18,19]. Historically it has received considerable attention due to its ability to grow in a variety of substrates such as carbohydrates, fatty acids and n-alkanes [7,20]. For example, in the decade of 1980 it was intensively used in the former USSR to produce single-cell protein as animal fodder from n-alkanes in fuel-oil distillates [7,21].

The industrial and biotechnological potential of *C. maltosa* motivated important research efforts to understand the biology of this yeast, especially during the 1990s and at the beginning of the 2000s. Hundreds of mutants were generated using physical and chemical mutagens, and several of its genes were cloned [20,22,24]. This culminated in host-vector systems that allowed its genetic manipulation despite the difficulties associated to the lack of a conventional sexual cycle that is typical of these fungi. However, only counted number of gene-mutants have been generated using these host-vector systems and the secondary effects of the physical and chemical mutagenesis used to generate the auxotrophies in these strains remain unclear. More recent work has focused on the capacity of *C. maltosa* to metabolize xylose and the genes required for this pathway [25,27]. Since it was considered a potential strain for industrial xylitol and ethanol production, the genome of *C. maltosa* was initially sequenced in 2013 using Illumina short-read technology. In addition, the genome draft of another strain sequenced using the same technology was made publicly available in 2023. Both drafts are considerably fragmented, consisting of thousands of contigs, and there was no publication associated with the first one where the genomic characteristics and methodology used for the assembly and annotation were described. There is no publicly available annotation for the draft that was deposited in 2023.

Despite the attention that *C. maltosa* attracted at the end of last century given its biotechnological potential, it has been mostly neglected as a comparative model to understand the pathogenicity of its closely related CTG species. Here, we re-sequenced the genome of *C. maltosa* using long-read technology, assembling a much more cohesive genomic draft and complete gene annotation. We also generated a set of strains and cassettes that allow its rapid genetic modification. As a proof of concept, we deleted both alleles of the *EFG1* gene which encodes a transcription regulator that controls processes associated to the virulence of closely related species. Even though *C. maltosa* is a non-pathogenic species inhabiting considerably different environments, characterization of the mutants showed that Efg1 is also involved in filamentation and biofilm formation. However, this transcription regulator seems to be a repressor of filamentation in this species. The genomic draft and genetic engineering tools developed here will allow using *C. maltosa* as a non-pathogenic reference to understand the molecular mechanisms implicated in the virulence of this medically important group of fungi. In addition, these tools will facilitate the use of this species for biotechnological applications.

## Methods

### Genome sequencing and assembly

High molecular weight genomic DNA was extracted from an overnight YPD culture of *C. maltosa* Xu316 [27] using a QIAGEN Genomic-tip 20 G^−1^ according to the manufacturer’s instructions. Genomic DNA was sequenced in parallel using the PacBio Sequel II platform and the DNBSeq platform at Novogene (Beijing, China) and BGI (Shenzhen, China), respectively. For PacBio long reads, three single-molecule real-time cells were used to generate a total of 586 673 raw reads with a length of between 30 and 52 kb and an estimated N50 value of 76.5 kb. Low-quality reads were filtered using Filtlong (v0.2.0) with the parameters ‘keep percent 80’ and ‘length weight 10’ [28]. For the DNBSeq PE150 short reads, over eight million raw reads were filtered by fastp (v0.23.2) using −c −x − y options for polyX trimming in 3′ ends, base correction in overlapped regions, and low complexity filtering, respectively [29]. The preliminary assembly was obtained using CANU (v1.8) with the high quality PacBio reads [30]. The clean DNBseq reads were then used in five consecutive rounds to polish the assembly with Pilon (v1.23) [31]. Genome completeness was evaluated with BUSCO v5.4.3 [32] using the lineage data set saccharomycetes_odb10. RepeatModeler 2.0.4 [33] was used to estimate the portion of the genome corresponding to repeat elements.

### Gene annotation

Gene annotation of the *C. maltosa* and *C. soja* genomes was performed using the MAKER pipeline (v2.31.8) [34]. This included an initial round of evidence-based alignment followed by two rounds of training and prediction using the SNAP and Augustus gene prediction programmes [35,36]. In the first round, we built a pangenome from the genomes of *C. albicans*, *C. tropicalis* and *C. parapsilosis*, and the resulting set of transcripts and proteins was provided as EST and protein evidence, respectively. In this initial round, as masking evidence, we also provided a custom repeat library generated with RepeatMasker v4.1.5 and RepeatModeler v2.0.4 [33]. The resulting gene models with proteins longer than 50 residues and AED scores below 0.25 were retained as evidence to train SNAP and Augustus for the two additional rounds of gene prediction. Augustus was trained with BUSCO using the initial HMM model of the fungi odb10 dataset. The functional annotation was performed using a blast homology search for each predicted protein sequence against the Uniprot database. Only the hits with an e-value inferior to 10E-6 were considered. Finally, InterProscan (v5.35.74) was used to detect protein domains, Gene Ontology terms and KEGG mappings from the InterPro database [37].

### Genomic comparison to closely related species

To compare the genome of *C. maltosa* with the ones of its closest related species, *C. tropicalis, C. sojae*, and *C. albicans,* we followed the workflow described by [38]. In short, this consisted in running Orthofinder (v2.5.5) [39] using the proteomes of the four species and using HMMER3 and Panther HMMs protein-coding gene family models [40,41]. To estimate significant gene family expansions or contractions across genomes we used CAFÉ (v5) [42]. For phylogenetic reconstruction, we ran Orthofinder with the proteomes generated here of *C. maltosa* and *C. sojae*, together with the 11 closest *Candida* species with available genomic annotations at GenBank, representatives of other CTG subclades (*Spathaspora passalidarum* and *Lodderomyces elongisporus)*, *C. auris* as an important pathogen and *S. cerevisiae* as an outgroup. The 569 single copy orthologs that were conserved in all the seventeen genomes were aligned using muscle (v3.8.31) [43] and the alignments were polished using TrimAl (v1.2) [44]. Finally, the concatenated alignments were used to reconstruct the phylogeny using RAxML-HPC2 on XSEDE (v8.2.12) in CIPRES [45] with the PROTCATWAG model and a total of 1000 bootstrap replicates [12].

To globally assess synteny between species, all-to-all BLASTP analyses of proteins were performed on protein sequences as well as within each species. BLASTP hits were filtered using an E-value≤10E-3. Then, all-to-all blastp results were used to identify syntenic regions between species using MCScanX [46] with the following parameters: minimum number of genes per syntenic block=5, E-value=10E-5, gap penalty = −1, maximum number of gaps=25, final score=50.

To identify enriched functional categories, Gene Ontology (GO) and domain information for each predicted protein sequence in the *C. maltosa* or *C. albicans* genomes were obtained using InterProScan v.5.66–98.0 with the Pfam library. This information was used to create a customized GO database for each species using the AnnotationForge R package [47]. GO enrichment analysis was done with the clusterProfiler R package [48], incorporating Benjamini-Hochberg multiple testing correction. Only categories with a q-value <0.05 were deemed statistically significant.

### Identification of genes associated with pathogenicity

Genes associated with pathogenicity were obtained from previously curated catalogues in *C. albicans* (33 genes from [49], 674 from [50] resulting in a set 629 genes) (Table S1, available in the online version of this article). *ALS, TLO* and *SAP* genes were obtained from Jackson *et al*., [3] and Oh *et al*., [51]. Genes related to filamentous growth were identified from the Candida Genome Database (http://www.candidagenome.org/) using the phenotypes section in tools utility, choosing the Phenotype Term ‘filamentous growth’ and including the following phenotypes: ‘filamentous growth: abnormal’, ‘filamentous growth: increased’, ‘filamentous growth: decreased’, ‘filamentous growth: absent’, ‘filamentous growth: decreased rate’ and ‘filamentous growth: delayed’ (Table S1). Orthologs between *C. albicans*, *C. tropicalis*, *C. maltosa* and *C. sojae* of the sets of genes mentioned above were defined from the genome-wide orthology assignments established before with Orthofinder. To verify the absence of pathogenicity-associated genes not found in *C. maltosa,* we conducted pseudogene predictions using Pseudopipe [52] on the genomic draft with default parameters, and the protein sequences of *C. albicans* as queries. Putative pseudogenes were filtered by excluding any matches that overlapped with functional gene annotations, transposon elements, or sequences shorter than 150 bp.

### Generation of auxotrophic strains

Given that *C. maltosa* is closely related to *C. tropicalis,* auxotrophic strains were generated using the SAT1-flipping strategy that has been optimized for *C. tropicalis* [53]. In brief, the SAT1-flipping module was used twice in consecutive rounds of transformation to delete each of the two alleles of the three amino acid genes, six tandem rounds of transformation in total. The module was removed after the deletion of each allele by expressing the *FLP* gene and thus inducing FLP-mediated, site-specific recombination. Deletion cassettes were generated by PCR with 99 bp primers that have ~70 base sequences that are identical to the 5′ and 3′ of the genes *HIS1*, *LEU2* and *ARG4* of *C. maltosa* (Table S2). Plasmid pEM018 was used as a template. The resulting cassette was purified with a QIAGEN MinElute PCR Purification Kit and ~0.5 µg were transformed by electroporation as previously described [53]. The *C. maltosa* strain transformed was Xu316 [27] (Table 1), a strain for which a genome draft was already available. Colony selection was done in YPD medium plates containing 400 µg ml^−1^ of nourseothricin (NAT) and the correct integration of the SAT1 cassette was verified by colony PCR of the 5′ and 3′ flanks. To recycle the SAT1 marker, previously to the second round of transformation, the cassette was flipped out by growing cells overnight at 30 °C in liquid YNB medium with 2 % casamino acids [53]. Flipped cells were subsequently screened for the loss of NAT resistance by replica plating in YPD medium plates supplemented with 400 µg ml^−1^ NAT. Apart from colony PCR of the flanks, the deletion of the second allele was also verified by the absence of the corresponding ORF through colony PCR with primers that anneal within the ORF, and by the inability of the strains to grow in SD media lacking the corresponding amino acid.

**Table 1.** Comparison of the genomic characteristics of the current (2024) and previous (2013 and 2023) *C. maltosa* genome drafts

Genomic features	2024 Draft	2013 Draft	2023 Draft
Number of scaffolds	45	2947	2349
Total size of scaffolds (bp)	14 040 896	12 826 280	15 987 543
N50 (bp)	1 315 209	11 156	11 900
Number of genes	5734	5986	na
Average gene length (bp)	1491	1715	na
Number of exons	8534	7042	na
BUSCO assembly completeness	98.9 %	97.7 %	93.9 %
BUSCO annotation completeness*	96.4 % (91.8 %)	92.8 % (89.6 %)	na

* Percentages in parenthesis are the completeness excluding fragmented genes.

### Reintegration of nutritional markers and deletion of target genes

To generate reference strains, we reintegrated the *C. albicans HIS1*, *LEU2* and *ARG4* markers into the triple auxotrophic strains, similarly to what has been done for the deletion of the two alleles of a gene of interest [53,54]. The reintegration cassettes were generated by fusion PCR of three fragments, ~300 bp sequences flanking the *C. maltosa* genes and the sequence coding for the * C. albicans* nutritional marker. The primers used are listed in Table S3. As a template to amplify the flanks we used genomic DNA of *C. maltosa,* and for the markers we employed plasmids pEM001, pEM002 and pEM003 for *HIS1, LEU2*, and *ARG4*, respectively [53]. The resulting cassettes were purified and concentrated using a QIAGEN MinElute PCR Purification Kit and ~0.5 µg were transformed by electroporation. Selection of transformants was performed by growth on SD plates without the corresponding amino acid. The integration in the correct locus was then verified by colony PCR of the 5′ and 3′ flanks. This resulted in the reintegration of the *C. albicans HIS1, LEU2* and *ARG4* nutritional markers in *C. maltosa* at the corresponding loci. The presence of the markers was finally verified by colony PCR with primers that target a region within the marker genes. The same strategy was followed for the deletion of the two alleles of *EFG1. C. albicans HIS1* nutritional marker was used to delete the first allele, and *LEU2* to delete the second, as has also been previously described [53,54].

### Filamentation essays

Filamentation was assessed as previously described [55]. Briefly, an overnight culture in YPD at 30 °C was washed twice with water and was subsequently resuspended in the inducing medium to a final OD_600_ of 1.5. The following media were tested: minimum medium (0.17 g Yeast Nitrogen Base without amino acids and ammonium sulphate, 0.5 g ammonium sulphate dibasic, 2 g glucose) adjusted at three different pH, 4.5, 6.0 and 7.0, RPMI medium (MP biomedicals, added with sodium bicarbonate and 2 % glucose), Lee medium [56,57], Synthetic dextrose medium (SD) [58], and synthetic defined medium supplemented with 0.75 % glucose and 50 % fetal bovine serum (FBS) [55,59]. Cells were inspected under an optical microscope with phase contrast at the time of induction and after 0, 3 and 6 h of incubation at 37 °C shaking in a roller drum. Micrographs were obtained using a Leica DMRX microscope using DIC and fluorescence of cells stained with calcofluor as previously described [60]. *C. maltosa* strains employed are described in Table 2, while for *C. tropicalis* we used CEM010 (WT) and CEM279 (*efg1*Δ/Δ) [59].

**Table 2.** *C. maltosa* strains used and generated in this study

Name	Species	Parent	Genotype	Source
Xu316	*C. maltosa*		*WT*	[27]
CMMC14	*C. maltosa*	Xu316	*leu2Δ::FRT/leu2Δ::FRT*	This Study
CMMC15	*C. maltosa*	Xu 316	*leu2Δ::FRT/leu2Δ::FRT*	This Study
CMMC27	*C. maltosa*	CMMC14	*his1Δ::FRT/his1Δ::FRT leu2Δ::FRT/leu2Δ:FRT*	This Study
CMMC28	*C. maltosa*	CMMC15	*his1Δ::FRT/his1Δ::FRT leu2Δ::FRT/leu2Δ:FRT*	This Study
CMMC45	*C. maltosa*	CMMC27	*his1Δ::FRT/his1Δ::FRT leu2Δ::FRT/leu2Δ::FRT arg4Δ::FRT/arg4Δ::FRT*	This Study
CMMC46	*C. maltosa*	CMMC28	*his1Δ::FRT/his1Δ::FRT leu2Δ::FRT/leu2Δ::FRT arg4 Δ::FRT/arg4Δ::FRT*	This Study
CMMC66	*C. maltosa*	CMMC45	*HIS1 Calb/his1Δ::FRT LEU2 Calb/leu2Δ::FRT arg4Δ::FRT/arg4Δ::FRT*	This Study
CMMC67	*C. maltosa*	CMMC46	*HIS1 Calb/his1Δ::FRT LEU2 Calb /leu2Δ::FRT arg4Δ::FRT/arg4 Δ::FRT*	This Study
CMMC55	*C. maltosa*	CMMC45	*his1Δ::FRT/his1Δ::FRT leu2Δ::FRT/leu2Δ::FRT arg4Δ::FRT/arg4Δ::FRT efg1Δ:: Calb HIS1/efg1Δ:: Calb LEU2*	This Study
CMMC56	*C. maltosa*	CMMC46	*his1Δ::FRT/his1Δ::FRT leu2Δ::FRT/leu2Δ::FRT arg4Δ::FRT/arg4Δ::FRT efg1Δ:: Calb HIS1/efg1Δ:: Calb LEU2*	This Study

### *In vitro* determination of biofilm formation

To assess biofilm formation, we followed the protocol previously described for other CTG species [61,62]. In brief, non-tissue culture treated six-well polystyrene plates were preincubated with adult bovine serum (BSA) overnight at 37 °C and shaking at 200 r.p.m. After removing the BSA and washing with phosphate buffered saline (PBS), 4 ml of fresh media were inoculated to an OD_600_ of 0.5 with cells from an overnight YPD culture. For adhesion, cells were incubated shaking at 200 r.p.m. and 37 °C for 90 min. Non-adhered cells were then aspirated, and the wells were washed twice with PBS to finally add 4 ml of the medium where biofilm formation would be assessed. After 48 h, unadhered cells were removed and biofilms were visually inspected. To determine the medium where *C. maltosa* formed thicker biofilms, we tested the following media: Lee medium [56,57], Lee medium supplemented with 1.25 % glucose, Spider medium with 1 % mannitol, glucose or xylose as carbon source, cornmeal liquid medium (prepared from cornmeal medium in cold water and filtering insoluble components before autoclaving) [63], and minimum media at pH 4.5. Based on the qualitative inspection of biofilms in the different media, we employed Spider with 1 % glucose to determine biomass dry weight of the *efg1* mutant. This was done by scraping and aspirating the 48 h biofilms onto a cellulose filter paper. Weight was determined in an analytical balance after drying biofilms for 24 h and subtracting the weight of a control well where cells had not been inoculated. Five replicate biofilms grown in separate wells were used for each strain. *C. tropicalis* strains used were the same as for the filamentation assays, while for *C. albicans* we used SN250 (WT) and TF156 (*efg1*Δ/Δ) [62].

## Results

### A cohesive *C. maltosa* genome assembly for comparative genomics and genetic engineering

As mentioned before, the currently available genomic sequences of *C. maltosa* were sequenced using short-read technology and the eight pairs of expected chromosomes are divided in over 2000 contigs. The considerably fragmented status of these genome drafts makes it difficult to perform thorough genome comparisons and design efficient genetic modification experiments. To achieve a more cohesive genome sequence of *C. maltosa* we performed long and short read sequencing using PacBio and DNBSeq technologies, respectively. Using the long reads, we tested three different genome assemblers (Canu, Falcon and Smartdenovo) to generate a new genome assembly. Based on the N50, L50 and NG50, the assembly made by Canu was selected and subsequently polished using the PE150 short reads. The resulting genome draft is composed of 45 contigs with a N50 of 1 315 209 bp. The size of the new genome assembly (14.04 Mb) was considerably larger than that of the first *C. maltosa* draft (12.8 Mb), but smaller than the latest sequence (15.9 Mb), while the GC content is very similar to both (~34 %). The portion of the genome that corresponds to repeat elements did not seem to explain the differences in genome size between the *C. maltosa* drafts since our assembly had most repeated elements (9.12 %) and it is not the largest sequence. Genome size and GC content were comparable to those of closely related species (*C. tropicalis* 14.3 Mb, 33 % GC, *C. sojae*, 15.1 Mb, 32 % GC and *C. albicans*, 14.3 Mb, 33 % GC). To assess completeness, we performed BUSCO analysis showing a coverage of 98.9 % that represents an improvement of more than 1 and 5 % from the previously available assemblies (Table 1).

The karyotype of *C. maltosa* was previously determined, showing eight pairs of chromosomes with a total size of close to 14 Mb [64]. Performing flow cytometry, we established that the strain that we are employing (Xu316) is diploid, as has been suggested for *C. maltosa* based on the ploidy of its closest relatives [65,66]. To better understand how the 45 contigs of our draft correspond to the eight chromosomes, we aligned them to the *C. tropicalis* and *C. albicans* genomes which are assembled at the chromosome level [67]. Twenty-seven contigs encompassing 13.63 Mb (97 % of the draft) were required to comprehensively cover the *C. tropicalis* and *C. albicans* chromosomes (Table S1) and a single contig was identified as the mitochondrial DNA (tig00000057). To concatenate the 45 contigs into chromosomes using these genomes as references we would need to assume that synteny has not changed in *C. maltosa*. However, comparison of the alignments revealed that the contigs did not align in the same order to both reference genomes and that single *C. maltosa* contigs aligned to more than one chromosome in the other two species. This probably reflects the large-scale differences in synteny of these species, as has been observed before [67]. Therefore, Hi-C or junction sequencing would probably be needed to assemble the *C. maltosa* draft at the chromosome level.

We also searched for the centromeres in the 45 *C. maltosa* contigs using the *C. tropicalis* and *C. albicans* sequences as seeds [67,68]. From all the centromeres of these two species, we were only able to find matches for five of the *C. tropicalis* sequences: CEN2 (tig00000043), CEN4 (tig00000039), CEN5 (tig00000001, tig00000034), CEN6 (tig00000034) and CENR (tig00000007). It is worth pointing out that contig tig00000034 aligned to two of the centromeres. This is in agreement with the repetitive nature of the centromere sequences which makes their assembly and incorporation into larger contigs difficult. Specific experiments to identify these regions would be needed as has been done for *C. tropicalis* and *C. albicans* [67,68].

To annotate the genes in the new *C. maltosa* genome assembly we built a reference pangenome from the genomic annotations of the three closest CTG species and whose genomes are well annotated*, C. tropicalis, C. albicans* and *C. parapsilosis*. Using this reference set of genes increased the possibility of identifying genes in the new genome by reducing the genes that are not present in the reference set. This approach predicted a total of 5734 genes composed of 8534 exons in the *C. maltosa* assembly (Table 1). Using an alternative gene prediction tool (YGAP) [69], we obtained a very similar number of genes (5781). The average gene length was 1491 bp and the average number of exons per gene was 1.47. BUSCO analysis of the annotated transcriptome using the Saccharomycetes database, showed a coverage of 96.4 %, which represented an improvement from the previously reported annotation (92.8 %) (Table 1). Analysis of the assigned functions of the absent genes in the previous annotation did not reveal enrichment of any particular functional category.

The genome of *C. sojae* has been recently sequenced using long read technology and it is almost assembled to the chromosome level [67]. However, there is no publicly available gene annotation that could be used to compare the gene content to other species, such as *C. tropicalis* or *C. maltosa*. Taking advantage of the pangenome built for the annotation of *C. maltosa* we also predicted gene models for the *C. sojae* genome. The resulting annotation is composed of 6100 genes which is closer to the 6441 genes of the latest *C. tropicalis* annotation. The genome of *C. maltosa* on the other side, had close to four and six hundred genes fewer than these genomes, respectively.

### Genes associated to pathogenicity are present in *C. maltosa*

**Fig. 2.**
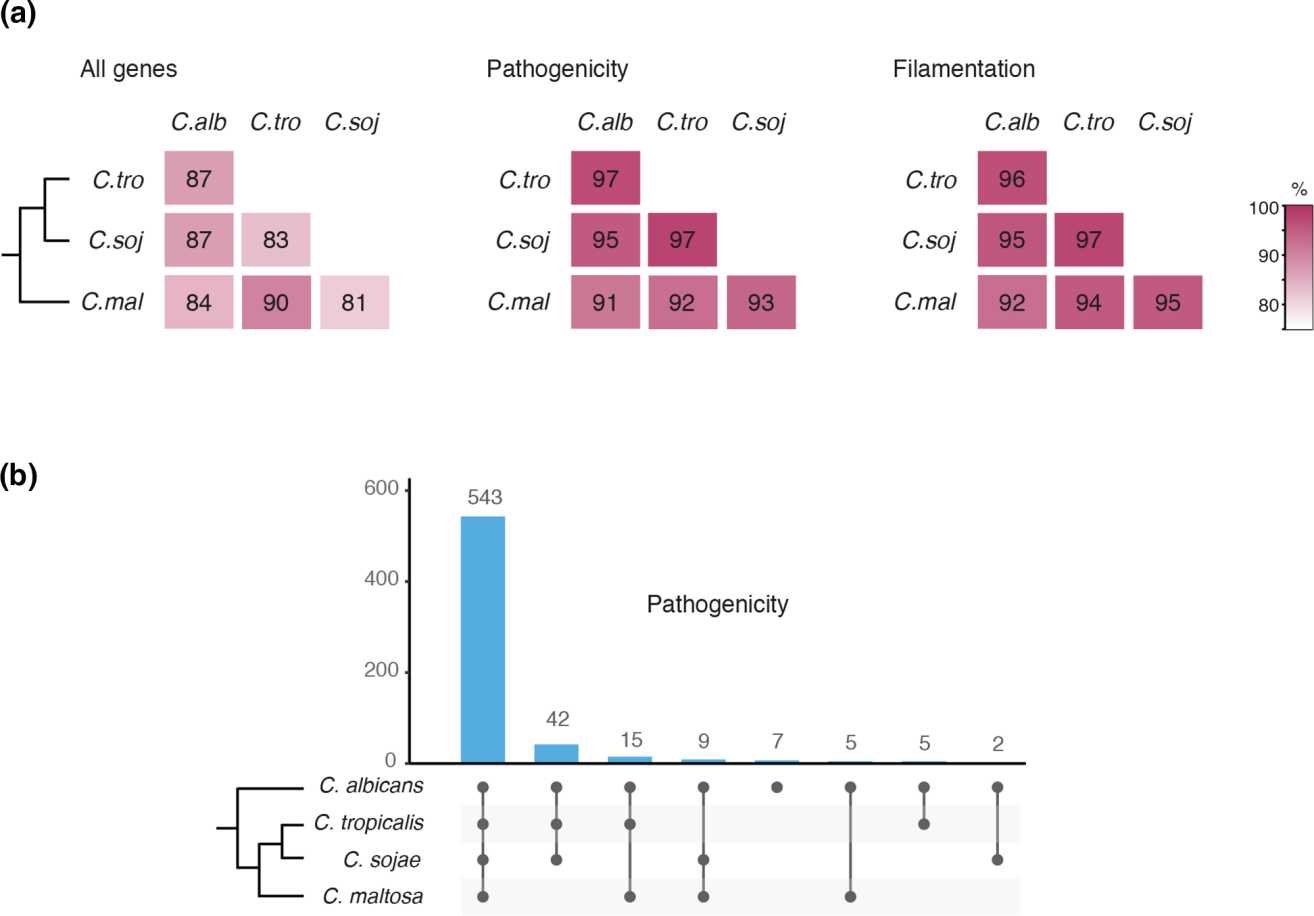
Most genes associated to pathogenicity are present in *C. maltosa.* **a**) Pair-wise comparison of shared orthologue genes between *C. maltosa* and its closest relatives. Each of the three heatmaps shows, from left to right, overall genes in the genome, pathogenicity associated genes and genes related to filamentation (see Methods). **b**) Bar plot showing the shared pathogenicity genes between the same four species, but in all possible comparisons. Most genes (86.4 %) are conserved in the four species analysed.

To identify genes that could be responsible for the differences in virulence between *C. maltosa* and closely related pathogenic *Candida* species, we first defined orthologous genes throughout the genome using OrthoFinder. We considered *C. tropicalis* as the closest species to *C. maltosa* that is human associated, and *C. albicans* as the best studied and most virulent species of the CTG clade (Fig. 1). *C. sojae* was also included in the analysis since it is as closely related to *C. maltosa* as *C. tropicalis*, but it is considered non-pathogenic (Fig. 1). As mentioned above, we needed to build the gene models for *C. sojae* since there was no publicly available annotation. We identified 4692 orthogroups that are shared between all four species from a total of 5739, and most of them are single copy orthogroups. *C. tropicalis* had the most unassigned genes (299), followed by *C. albicans* (192), *C. sojae* (141) and *C. maltosa* (91) (Fig. 2). In agreement with its smaller genome, *C. maltosa* had the largest number of gene family contractions between these species (34 contractions). In terms of synteny, of the 5734 genes of *C. maltosa*, 4454 (77.67 %), 4448 (77.57 %) and 4641 (80.9 %) were grouped in 295, 330 and 293 syntenic clusters for * C. tropicalis*, *C. sojae* and *C. albicans*, respectively.

To identify pathogenic associated genes among the set of orthologs in these species, we used two previously curated gene catalogues [49,50]. These lists include 629 genes of *C. albicans* that have been implicated in different aspects of pathogenicity such as hyphae and biofilm formation, adhesion, evasion of the immune system and invasion of the host (Table S1). Of these genes, 96.6 % have an ortholog in *C. tropicalis*, 91.4 % in *C. maltosa* and 94.9 % in *C. sojae* (Fig. 2). The percentage of shared genes in this category is more than the proportion of overall paralogs between *C. albicans* and these three species (87, 84, and 87 %, respectively) and is more than what would be expected by chance (< 30 %).

We also assessed gene conservation in other specific gene families that have been linked to virulence in these species, namely telomere-associated genes (*TLOs*), agglutinin like-sequences (*ALSs*), secreted aspartyl proteinases (*SAPs*) and filamentation genes (Table 3 and Fig. 2). TLOs are a family of genes that have a conserved N-terminal Med2 domain and therefore, apart from their role in virulence, are thought to be related to general transcription [13,70]. *C. albicans* has 15 *TL*O genes [3], while only one was present in *C. maltosa.* However, *C. tropicalis* and *C. sojae* also had only one *TLO* ortholog, suggesting that the ancestor of these three species most probably also had one, and the number is not related to their ability or lack thereof to colonize humans. The synteny of the *TLO* gene in *C. maltosa* is conserved all the way to *C. albicans*. The *ALS* genes are central for cell adhesion, tissue invasion and biofilm formation [51,71]. *C. albicans* is known to have nine of these genes [3] and 16 have been found in *C. tropicalis* [71]. We found eleven *ALS* orthologs in *C. maltosa* and 13 in *C. sojae*. Synteny conservation for this family of genes is harder to assess since it is difficult to establish one-to-one orthologs. The three *ALS* from *C. maltosa* where orthology could be established have the same adjacent genes in *C. tropicalis* and *C. sojae* and only the synteny of one is preserved in *C. albicans. SAPs* have also been associated to adhesion but their role in virulence is most probably related to host tissue invasion through their hydrolytic activity [72,73]. *C. albicans* has ten different *SAPs* and *C. tropicalis* six [3], while the species not associated to humans had ten and six genes in the case of *C. maltosa* and * C. sojae,* respectively (Table 3). From the nine *SAP* genes in *C. maltosa* for which one-to-one orthology could be established, all had conserved synteny in *C. tropicalis*, and six in *C. sojae* and eight in *C. albicans.* In addition, given the importance of filamentous growth for the biology of these species, a set of 447 genes related to hypha formation in *C. albicans* according to CGD was used to identify orthologous genes in *C. maltose* and related species. Almost all of these genes have an ortholog in the other three species, although the set is overall less conserved in *C. maltosa* (96.2 % in *C. tropicalis*, 95.3 % in *C. sojae* and 92.3 % in *C. maltosa*) (Fig. 2).

**Table 3.** Number of *ALS, TLO* and *SAP* genes in *C. maltosa* and its three closely related species

Species	*ALS*	*SAP*	*TLO*
*C. albicans*	9	10	15
*C. maltosa*	11	10	1
*C. sojae*	13	6	1
*C. tropicalis*	16	6	1

To verify that the pathogenicity and filamentation associated genes (seven and nine genes, respectively) detected as missing in *C. maltosa* are absent from the genome, we searched for vestiges of these ORFs employing a pipeline to detect pseudogenes (see Methods). Of the 16 genes, only two were identified as pseudogenes, strongly suggesting that the observed absences are actual gene losses. This approach also revealed that *C. maltosa* has overall a considerably larger number of pseudogenes compared to its sister species. Taking into account only pseudogenes that do not overlap with annotated features, 188 were identified in *C. maltosa*, while 45, 66 and 120 were found in *C. tropicalis*, *C. sojae* and *C. albicans*, respectively. This suggests a possible mechanism through which the genome of *C. maltosa* may have contracted, although it did not particularly affect genes associated to pathogenicity. Instead, there were four other functional categories enriched among the genes missing in *C. maltosa* and present in the other three species: ‘signalling’, ‘signal transduction’, ‘cell communication’ and ‘structural constituent of cell wall’.

### A set of *C. maltosa* auxotrophic strains for its genetic modification

As most of its closely related species, *C. maltosa* has only been isolated in a diploid state and it has not been observed to undergo a sexual cycle. For these reasons, to generate a gene knockout strain it is necessary to directly delete the two alleles of the gene of interest. To this end, we generated auxotrophic strains for three nutritional markers in *C. maltosa*, leucine, histidine, and arginine. Two of the markers can be used to delete the two alleles of the targeted gene and the third for reintegration of a copy for complementation assays. This strategy has been routinely used in other related *Candida* species, even for the generation of large collections of knockout strains [50,74]. In addition, auxotrophic strains have been instrumental in implementing CRISPR based strategies in related species [75]. To generate the triple auxotrophic strains, we tandemly deleted the two alleles of the genes *LEU2*, *HIS1* and *ARG4* using the SAT-1 flipping system that has been optimized for *C. tropicalis* [53]. The flipping strategy was an effective method to genetically modify *C. maltosa*, although considerably laborious given that the *SAT1* marker must be flipped out between every round of allele deletion and due to the incubation time needed for the cells to express the resistance marker before selection can be applied. These factors can be a considerable disadvantage for constructing collections of mutant strains when compared to the strategy that uses multiple nutritional markers [53,54].

The ORFs that corresponded to the *LEU2*, *HIS1* and *ARG4* genes in *C. maltosa* were identified as the best blast reciprocal hits to the *C. tropicalis* and *C. albicans* genes. The protein sequence identity of the three genes among the three species ranged from 91.69 to 95.44 % and at the nucleotide level it went from 84 to 85 %. The differences at the nucleotide level permitted using the genes of *C. albicans* as the nutritional markers so that they are not inserted at the endogenous *LEU2*, *HIS1* and *ARG4* loci, but rather at the locus that is being targeted for deletion. On the other side, the conservation at the protein level ensured functional complementation by the *C. albicans* markers. Two independent triple auxotrophic strains were generated in the genetic background for which a genomic sequence was previously available, Xu316 [27] (Table 2). In addition, in the process of generating these strains, single leucine auxotrophic strains and double leucine and histidine auxotrophic strains were generated. All these strains are a valuable resource for the genetic modification of *C. maltosa*.

To generate a set of reference strains we also reintegrated the amino acid markers of *C. albicans* at the endogenous *LEU2*, *HIS1* and *ARG4* loci (Table 2). To this end, deletion cassettes were constructed with flanking sequences identical to the up- and down-stream region of the amino acid genes. Growth of colonies in medium lacking the corresponding amino acid was used to select for transformants showing that all amino acid genes from *C. albicans* are able to functionally complement the auxotrphy in *C. maltosa*. The correct integration of the markers was finally verified by colony PCR with primers directed to the junctions of the markers and the regions surrounding the *LEU2*, *HIS1* and *ARG4* loci. The resulting set of strains (Table 2) are the direct references for the phenotypic characterization of knockout mutants generated using the genetic modification strategy here implemented.

### *EFG1* has a role in filamentation and biofilm formation in *C. maltosa*

To test the gene knockout strategy directly with a gene of interest, we deleted the ortholog of the gene encoding for the transcription regulator Efg1. In closely related species, Efg1 regulates several morphological transitions [76,79] but it is not an essential gene, being an ideal candidate for the proof of concept. The ORF of *EFG1* in *C. maltosa* was identified during the annotation of the genomic sequence and it was independently verified as the best reciprocal blast hit with the gene of * C. tropicalis*. The overall sequence identity at the protein level between the genes of these two species is 67.01 % and its synteny is conserved. Gene order is also conserved around *EFG1* in *C. sojae*, but not in *C. albicans* as has been reported before [59]. For its deletion, *C. albicans LEU*2 and *HIS1* cassettes were employed, as described for the generation of the reference strains. These cassettes were used to tandemly delete the two alleles of *EFG1,* and the knockout was successfully verified by colony PCR. Our results showed that the strategy is an efficient way to generate gene knockouts as has been done in other *Candida* species [53,54].

**Fig. 3.**
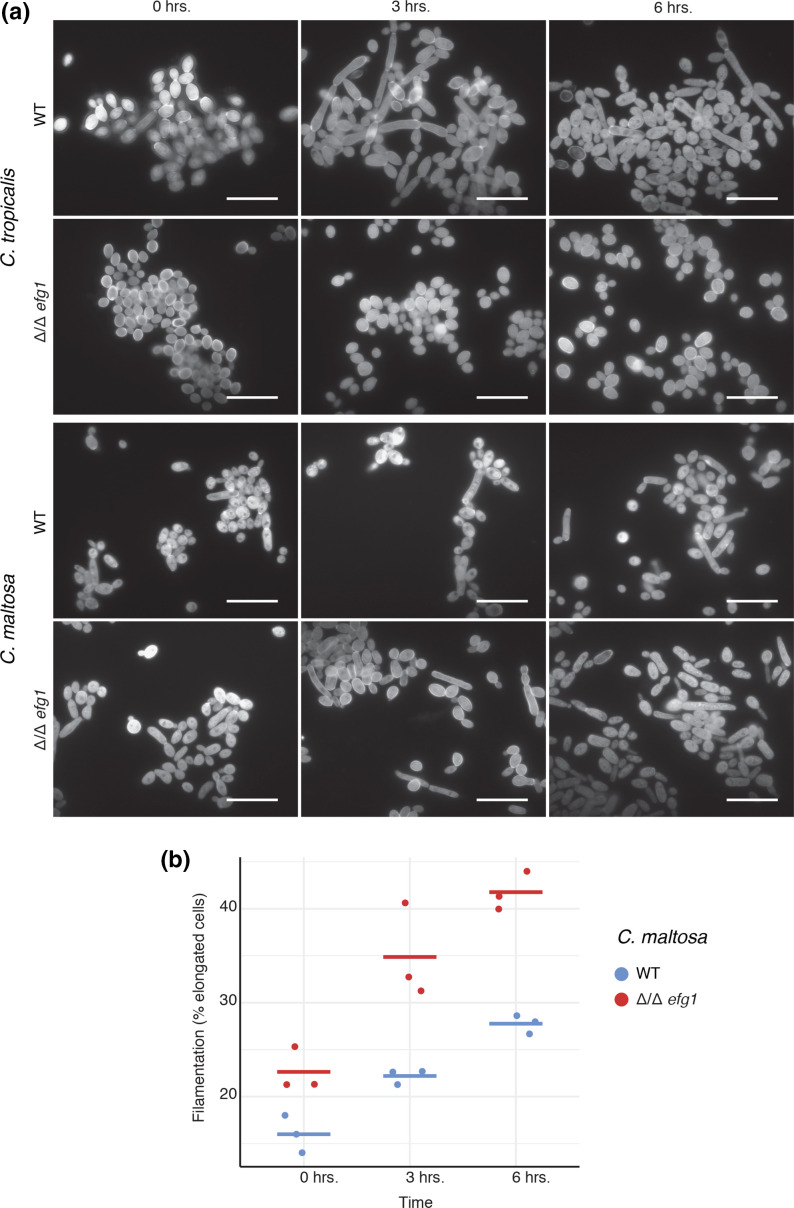
*EFG1* is involved in filamentation in *C. maltosa.* **a**) Micrographs of *C. maltosa* and *C. tropicalis* and its corresponding *Δ/Δefg1* mutants under filamentation inducing conditions (minimum media pH 4.5). Representative micrographs are shown at the time of transfer to the induction medium (0 h) and after 3 and 6 h of culture in these conditions. Scale bar represents 20 µm. **b**) Quantification of the fraction of elongated cells of *C. maltosa* after transfer to inducing conditions through time. The *t*-test *P* values were 0.0205, 0.0455 and 0.0021 for the three time points respectively.

To assess whether the function of Efg1 is conserved in *C. maltosa,* we tested the gene deletion mutant for its ability to filament and form biofilms. These two morphological transitions depend on this transcription regulator in *C. tropicalis*, *C. albicans* and other related species [59,61]. To this end, standard *in vitro* filamentation and biofilm formation assays were employed, although a variety of media were tested to identify the optimal condition for this species. For filamentation, minimum medium had been previously used for *C. maltosa* [80] and it was where we observed that it filamented the most. However, despite the six different media tested, *C. maltosa* did not filament as efficiently as *C. tropicalis* and *C. albicans* in liquid cultures. In addition, *C. maltosa* did not form hypha and the cells that underwent a morphological transition only elongated into what could be pseudohyphal growth. This is in agreement with previous work with other *C. maltosa* strains in which only pseudohyphae formation has been observed [80,81]. When assessing the *efg1* mutant, surprisingly, we observed a 30 % increase in filament formation after 6 h in the inducing conditions compared with the reference strain (Fig. 3, *P*=0.0021, *t*-test). This filamentation increase contrasted the phenotype observed in *C. tropicalis*, in which the *efg1* mutant did not filament under the conditions tested. However, the deletion of *EFG1* has been previously associated to increased pseudohyphal growth in *C. parapsilosis* [82].

**Fig. 4.**
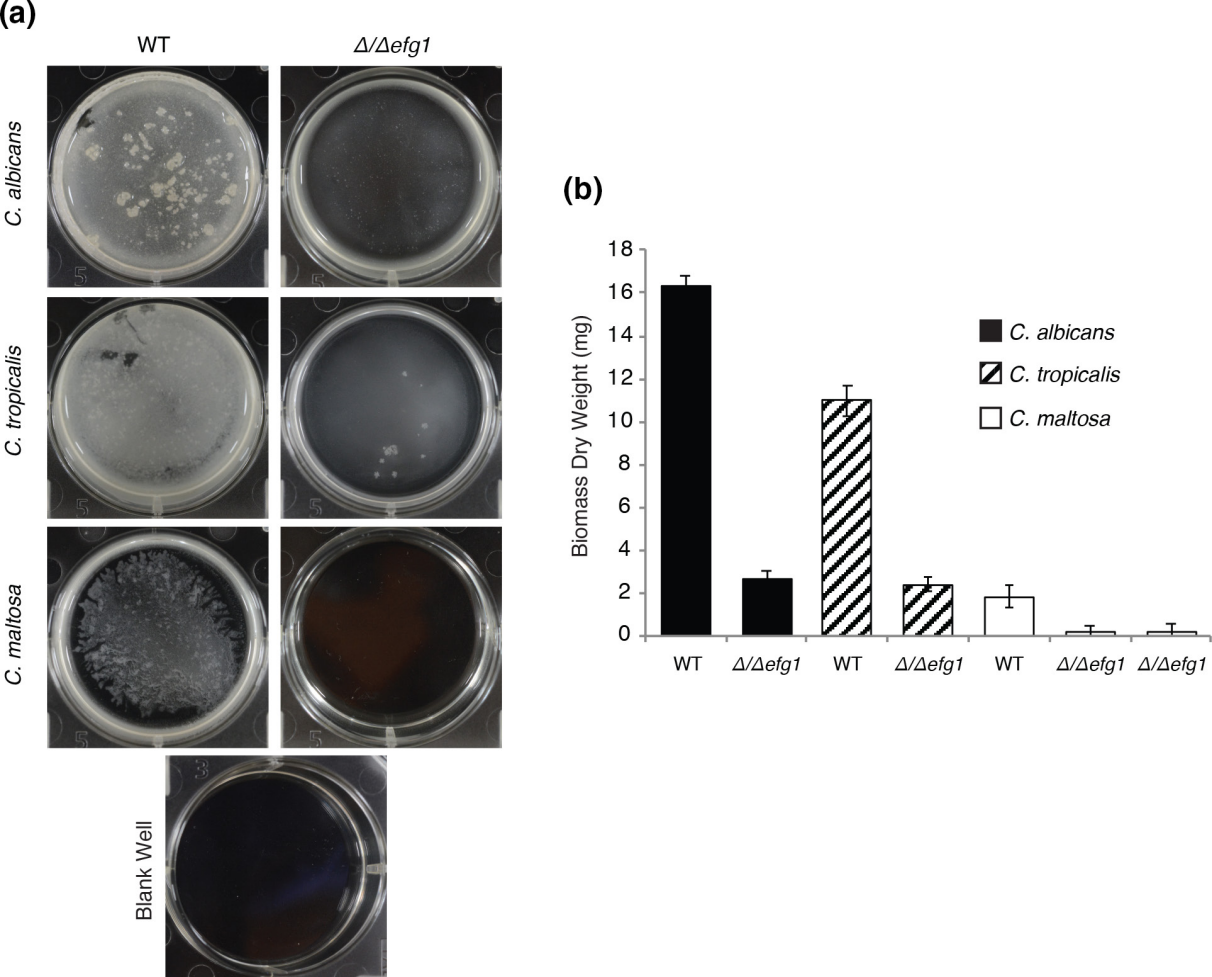
*EFG1* is required for biofilm formation in *C. maltosa.* **a**) Biofilm formation by *C. maltosa, C. albicans* and *C. tropicalis,* and their corresponding *Δ/Δefg1* mutants at the bottom of six-well polystyrene plates in Spider glucose medium (see Methods). One well is shown per strain. **b**) Comparison of the biomass dry weight of the biofilms. Dry weight of five replicates was used to determine the standard deviation. The *t*-test *P* values were 3.9×10^−6^, 0.0001, 0.0013 and 0.0013 for the comparison of the mutant and wild-type strain of *C. albicans*, *C. tropicalis* and the two mutant strains of *C. maltosa,* respectively.

For biofilm formation, performing qualitative assays we determined that Spider medium or Spider replacing mannitol by glucose as a carbon source, were the media where *C. maltosa* was better able to form biofilms. However, as for filamentation, in all media tested, the biofilms formed by *C. maltosa* were considerably thinner than those formed by *C. tropicalis* or *C. albicans*. To test biofilm formation in the *efg1* mutant we employed Spider with glucose since this medium has been shown to also be optimal for the sister species *C. tropicalis* and would therefore facilitate comparisons [61]. Although the biofilms formed by wild-type *C. maltosa* are relatively thin, we did observe a close to 90 % reduction in the dry-weight biomass of the biofilms formed by the *efg1* strain (Fig. 4, *P*=0.0013, *t*-test). We also assessed biofilm formation in minimum medium since it was where this species filamented more efficiently. The results showed a similar trend, the *efg1* mutant formed considerably thinner biofilms and overall *C. maltosa* formed thinner biofilms than *C. tropicalis* and *C. albicans*. Together with the filamentation phenotypes observed, these results showed that the overall function of Efg1 as a morphological regulator is conserved in *C. maltosa*.

## Discussion

Due to its phylogenetic position and avirulence, *C. maltosa* is a promising species to understand the mechanisms that underly the pathogenicity of closely related *Candida* species through comparative studies. Here, we generated a comprehensive genomic sequence and constructed a set of strains that will facilitate the genetic manipulation of *C. maltosa*. Although our genome draft is considerably more cohesive than previous drafts, it is still not assembled at the chromosome level. At this point, further experimental work would probably be needed to concatenate the contigs into chromosomes. Analysis of the genomic draft and its annotation revealed that *C. maltosa* has several hundred fewer genes than its pathogenic relatives *C. tropicalis* and *C. albicans*. Thorough computational scrutiny with alternative approaches strongly suggested that these are actual gene losses and not the result of problems in the genome assembly. The gene reduction does not seem to be specific to non-pathogenic species since * C. sojae*, a closely related non-virulent species, also has considerably more genes than *C. maltosa*. In addition, the missing genes are not particularly enriched for genes related to pathogenicity. In fact, *C. maltosa* has orthologs of most of the genes that have been previously associated to virulence in *C. albicans*. Gene loss in yeasts has been associated to tolerance of varied stresses [83,84], which could be the conditions that *C. maltosa* is encountering in the industrial environments where it has been isolated.

The case of the *TLO* genes is worth pointing out. These genes encode a conserved Med2 domain that is a component of the Mediator complex, with a possible role in the expression of genes related to virulence such as those involved in antifungal drug resistance and dimorphism [13,70]. *C. albicans* has an unusually high number of *TLO* genes (15), while *C. maltosa*, *C. sojae*, and even the pathogenic *C. tropicalis*, have only one. *C. dubliniensis*, the closest species to *C. albicans*, has two orthologs suggesting that the large number of *TLOs* in *C. albicans* could represent gains associated to the ability of this species to colonize the human body, but that are not shared with other pathogenic species. Despite the specific cases, overall, the dissimilarities in virulence between these *Candida* species does not seem to be easily explained by broad changes in gene content. Instead, other evolutionary modifications such as those involving the regulation of genes may be more important underpinnings of these differences. Although further investigation is needed, the specific lower number of genes observed in *C. maltosa* seems not to be related to its inability to colonize the human body.

The auxotrophic strains that we generated here, together with previously available amino acid marker cassettes [53], allowed the implementation of an effective strategy to genetically modify *C. maltosa*. Previously developed host-vector systems for this species relied on auxotrophic strains generated by random mutagenesis screens [20,23] with the risks of generating off-target mutations that these approaches have. In fact, high instability of some of these mutants has been observed [85]. Taking advantage of the new genome assembly and a SAT-FLP cassette developed for *C. tropicalis*, we were able to specifically target three amino acid genes that have been typically employed to produce auxotrophies for the generation of gene deletion mutants in several related species. Two independent auxotrophic lines were generated so that deletions can be performed in replicates. Apart from using the triple auxotrophic strains for gene deletion as performed here, these strains could be the basis to implement CRISPR-Cas9 approaches in this species. For example, there is a CRISPR system developed for *C. albicans* that relies on the integration of the cassette in the *LEU2* gene of *C. maltosa* [75]. This gene has been used as a complementation marker in large gene knockout efforts in *C. albicans* [54]. In principle, using the strains we have generated here, this system could be easily implemented to perform genetic modifications in *C. maltosa*.

As a proof of concept of the genetic modification approach, we successfully generated a homozygous gene knockout of the *EFG1* gene. In related CTG-species, this gene encodes a transcription regulator that controls several cellular transitions including filamentation, biofilm formation and white-opaque switching [59,76]. All of these morphological processes have been associated to the subsistence of the pathogenic species in the human host and the balance between commensal and pathogenic states [77,86]. The involvement of *EFG1* in the regulation of some of these morphological transitions such as filamentation and biofilm formation, has been shown to be conserved as far as in *C. parapsilosis*. In agreement, disruption of *EFG1* in *C. maltosa* led to phenotypic changes in filamentation and biofilm formation. Interestingly, contrary to the observation in *C. tropicalis*, the *efg1* mutant showed increased filamentation capacity in the conditions here employed. These results agree with previous observations in *C. parapsilosis*, in which Efg1 could be operating as a negative regulator of filamentation [82]. Our results also suggested that the defect in biofilm formation of the *C. maltosa efg1* mutant does not fully depend on a negative effect in filamentation.

Despite the conservation in the involvement of *EFG1* in these morphological transitions, what is clearly different is the reduced ability of *C. maltosa* to filament and form biofilms as the ones formed by the pathogenic species [61]. In our filamentation assays, this species only formed elongated cells and actual hypha were never observed. This is in agreement with previous work using different *C. maltosa* strains, in which only pseudohypha have been reported [80,81]. In the *in vitro* assay that we have routinely used, the biofilms formed by *C. maltosa* are considerably lighter and less structured. It is possible that this species can filament or form biofilms under different conditions, although we did test several media including some that resemble the conditions where *C. maltosa* has been isolated. These observations suggest that *C. maltosa* is overall a poorer filament and biofilm former or that it establishes these morphological structures under conditions that are considerably different than those where the pathogenic species form biofilms.

Research involving *C. maltosa* has decelerated in the last ten to fifteen years, with the focus that this species received by research groups mainly in Japan and Europe during the nineties and at the beginning of the current century [7,24, 80] seeming to have dissipated. We propose using this species as a comparative model to better understand the molecular basis of the virulence of closely related *Candida* species, especially *C. tropicalis*. The genomic draft and genetic modification strategies presented here will allow comparisons of, for example, the regulatory mechanisms that control filamentation and biofilm formation. Complementation assays with the orthologs of *EFG1* could reveal whether changes in the coding region or the way this transcription regulator is expressed are responsible for the observed phenotypic differences. Furthermore, experiments targeted to the genes that are absent in *C. maltosa* and *C. sojae,* the non-virulent species, could be very informative regarding the molecular underpinnings of pathogenicity in these fungi. In addition to these contributions, these tools have the potential to facilitate research towards biotechnological applications with *C. maltosa*.

## supplementary material

10.1099/mic.0.001447 Uncited Table S1.

## Abbreviations

BSA: adult bovine serum
FBS: fetal bovine serum
GO: gene ontology
ITS: internal transcribed spacer
PBS: phosphate buffered saline
RPMI: Roswell Park Memorial Institute
SD: sinthetic defined
YPD: yeast extract peptone dextrose
